# Is humane slaughtering of rainbow trout achieved in conventional production chains in Germany? Results of a pilot field and laboratory study

**DOI:** 10.1186/s12917-020-02412-5

**Published:** 2020-06-15

**Authors:** Verena Jung-Schroers, Uta Hildebrandt, Karina Retter, Karl-Heinz Esser, John Hellmann, Dirk Willem Kleingeld, Karl Rohn, Dieter Steinhagen

**Affiliations:** 1 Fish Disease Research Unit, Institute for Parasitology, University of Veterinary Medicine Hannover, Bünteweg 17, D-30559 Hannover, Germany; 2 Institute of Zoology, University of Veterinary Medicine Hannover, Bünteweg 17, D-30559 Hannover, Germany; 3 Present address: Landesamt für Natur, Umwelt und Verbraucherschutz Nordrhein-Westfalen (LANUV), Fisheries Ecology, Heinsberger Straße 53, D-57399 Kirchhundem-Albaum, Germany; 4 Lower Saxony State Office for Consumer Protection and Food Safety, Veterinary Task-Force, Eintrachtweg 19, D-30173 Hannover, Germany; 5 Institute for Biometry, Epidemiology, and Information Processing, University of Veterinary Medicine Hannover, Hannover, Germany

**Keywords:** Rainbow trout, Conventional production, Stunning procedures, Animal welfare, Visually-evoked responses

## Abstract

**Background:**

Rainbow trout, *Oncorhynchus mykiss*, is an important fish in European freshwater aquaculture. This industry sector is dominated by small family-owned enterprises located in rural areas. A large percentage of rainbow trout produced by these small enterprises is marketed directly and killed on demand and not processed in commercial processing plants. EU and national regulations stipulate that fish shall be stunned prior to killing and slaughter. The overall objective of this study was to monitor how stunning interventions were integrated into the production chains of German conventional trout aquaculture in order to safeguard animal welfare during stunning and killing. For this, the stunning and slaughtering processes were monitored on 18 rainbow trout farms in various German federal states. During the on-farm research, (i) the stunning success, (ii) injuries related to the stunning procedure, (iii) duration between stunning and killing, and (iv) visible responses at the time of slaughtering were assessed as welfare indicators. In addition, haematological and biochemical blood parameters were measured as indicators for physiological stress. Due to the fact that stunning interventions should induce a loss of consciousness in fish, in a laboratory study, it was examined whether the absence of the brainstem/ behavioural responses, opercular movements (OM) or eye-rolling reflex (vestibulo-ocular reflex, VOR) was correlated with the stage of insensibility.

**Results:**

The majority of rainbow trout farms applied manual percussion (38%) or electrical stunning (48%), while on 14% of the farms, the fish were stunned by electrical stunning which was immediately followed by manual percussion. After percussive stunning, about 92.3% of the rainbow trout displayed no OM or VOR as brainstem/ behavioural indicators of consciousness. This percentage varied on farms which applied electrical stunning. While on the majority of farms, 95 to 100% of the fish were unconscious according to the observation of brainstem/ behavioural indicators, the stunning intervention was less effective on farms where rainbow trout were stunned at current densities below 0.1 A dm^2^ or for a few seconds only. The laboratory study confirmed that the absence of brainstem/ behavioural indicators correlated with the absence of visually evoked responses (VER) of the brain to light stimuli as a neuronal indicator of insensibility. Therefore, the brainstem/ behavioural signs can be used to interpret the stage of insensibility in rainbow trout. A stage of insensibility could safely be induced by exposing portion-sized rainbow trout to an electric current density above 0.1 A dm^2^. This was not influenced by the orientation of the electric field.

**Conclusions:**

In conventional aquaculture, rainbow trout can effectively be stunned by manual percussion or electrical stunning. Consciousness can be monitored by the absence of opercular movements or the eye-rolling reflex, which are lost approximately at the same time as neurological responses like VER. For safeguarding animal welfare during stunning and killing of rainbow trout in conventional production processes, the stunning process requires careful attention and the operating personnel need to be trained in using the stunning devices and recognising indicators of consciousness.

## Background

Fish is widely accepted as a healthy food and the growing demand for fish has led to an overexploitation of wild fishery stocks and a rapid development of the production of fish in aquaculture [1, 2]. Today, aquaculture contributes the major share of globally consumed fish [1]. In contrast, in the EU, aquaculture accounts for only 20% of the total fish production, but the sector is expected to rise in order to meet the demands of the European market for fish. The European aquaculture industry is very diverse and produces cold water marine fin fish species in the North Atlantic, warm water marine fin fish species in the Mediterranean and various fresh water species [3]. Freshwater aquaculture is well established in European fish farming, and one of the major farmed freshwater fish species is rainbow trout, *Oncorhynchus mykiss*. It is raised in Europe mainly in Norway, Italy, Denmark, France and Poland but it is also the most important fish species grown in German aquaculture. While the European marine cold- and warm-water aquaculture sub-sectors include large multi-national companies and the consolidation and vertical integration continue in this industry, the freshwater aquaculture sector is dominated by family-owned small and medium (SME) or micro-enterprises which are located in coastal and rural areas and contribute significantly to the economy of these areas [4].

European consumers expect that the production of fish in aquaculture in Europe respects sustainability, complies with consumer protection standards and that animal welfare is safeguarded during production and the time of slaughtering [2, 4, 5]. In addition, cognitive and mental capacities of fish were subject of various scientific studies and review articles published recently [6–12]. However, vertebrate organisms, commonly called “fish” account for more than 60% of the known vertebrate species and are adapted to various aquatic habitats. The cognitive and mental capacities were studied in a small number of species only (see [6]). These studies, however showed that several teleost fish species respond to stressful situations and painful stimuli by behavioural and physiological reactions [7, 9, 13, 14]. Furthermore, in several finfish species observational and social learning was documented (cited from [6]), as well as long-term memories and the development of complex traditions [7, 10]. These cognitive and learning capacities of fish are used as indicators for consciousness and pain perception [6, 7, 10, 13, 15] and are regarded as important for the ethical treatment of fish [7, 16].

In Europe, fin fish are included in the European convention of the protection of animal kept for farming purposes (treaty 87) [17], the European convention of the protection of animal for slaughter (treaty 102) [18], and the Council Regulation (EC) 1099/2009 [19] “on the protection of animals at the time of killing”. However, with regard to fin fish, only the requirements stipulated in Article 3 (1) apply, according to which animals shall be spared any avoidable pain, distress or suffering during their killing and related operations. Further requirements regarding the killing or slaughtering of fin fish derive from national regulations within EU member states. For example, the German Animal Welfare Act [20] and the German Regulation for Animal Welfare during Slaughter [21] stipulate that fin fish shall be stunned prior to killing or slaughter. A survey on stunning and killing methods applied in different aquaculture production chains for freshwater rainbow trout in European countries revealed that mainly electrical stunning was used by farms and abattoirs, but also manual percussion and asphyxia in ice slurries were applied [4]. When the European Food Safety Agency (EFSA) analysed methods for stunning and killing freshwater rainbow trout, in particular, the application of asphyxia in ice slurries, asphyxia at ambient temperatures or exposure to CO_2_ were regarded as resulting in poor animal welfare, whereas percussion and electrical stunning were assessed as reliably causing unconsciousness in the vast majority of the examined fish and meeting animal welfare standards [22].

Stunning interventions which safeguard animal welfare should reliably induce a loss of consciousness, i.e. a stage of insensibility in fish, which continues until the fish can be humanely killed by exsanguination. In order to establish whether a stunning intervention induces a loss of consciousness, indicators have to be defined which allow to recognise whether the fish is in a stage of insensibility. Since consciousness implicates the ability of organisms to perceive and thereby respond to selected features of their own environment [6, 22], this can be assessed by recording brain activity in form of an electroencephalogram (EEG). In particular, sensory evoked-responses like visually evoked-responses (VER) indicate that the brain can respond to an external sensory stimulus [23]. In the state of unconsciousness, animals display abnormal EEGs [24] and it can be assumed that the respective brain cannot process an external sensory stimulus [23]. Hence, a VER could not be recorded from an unconscious animal [23]. Neurological investigations of consciousness by EEG recordings, however, are only suitable for laboratory studies and cannot be routinely applied on farms. Nonetheless, brainstem/behavioural indicators like control of body posture, regular opercular movements (indicating breathing activity) or eye-rolling reflex (vestibulo-ocular reflex, VOR) may be used to interpret stages of consciousness on farms. These indicators, however, can be misleading due to different tolerance levels or behavioural repertoires in different fish species. Therefore, they can be used only in those fish species in which these indicators correlate with neuronal activity [25]. In rainbow trout recovering from an electrical stunning intervention, Kestin et al. [26] documented that prior to the return of rhythmic opercular movements, the brain showed an epileptiform activity pattern. On the basis of this observation, it was assumed that rainbow trout are insensible when eye-rolling and breathing reflexes are absent [27]. Therefore, it can be assumed that on farms the observation of the eye-rolling or breathing reflexes may be used to determine whether rainbow trout are insensible [27].

In conventional production chains of German rainbow trout aquaculture, about 50% of rainbow trout are marketed directly and killed on demand. Then, percussive stunning is done manually, one at a time, by one by applying a sharp blow to the fish’s head with a wooden or plastic club. In accordance with EFSA [22], this achieves immediate stunning when done properly. A mishit or a hit with insufficient force, which does not render the fish unconscious, would result in the fish being processed while still conscious when insufficient stunning is not recognised by the operator and further stunning is not applied. In a risk assessment, EFSA experts considered this as a major hazard associated with percussive stunning [22]. As an alternative to percussive stunning, electrical stunning is applied, mainly by passing an electric current through fish immersed in tank water by means of submerged electrodes which cover the whole area of two opposite walls of the stunning tank. Alternatively, fish can be stunned after being removed from the water; this is called dry stunning in the industry. Then fish are placed on a device which allows an electric current to be passed through the head of the fish outside of the water. In both cases, applying the electric current renders the fish unconscious instantaneously [22]. Hazards to fish welfare related to electrical stunning include applying an electric current of insufficient current density for rendering the fish unconscious or applying the electric current for a short period of time only, which allows the fish to recover from stunning before being killed. This also would result in processing the fish while conscious and impair fish welfare [22].

Currently, no data are available on how animal welfare is safeguarded in German trout aquaculture during stunning and killing of farmed rainbow trout. The German Regulation for Animal Welfare during Slaughter [21] stipulates the use of percussive or electrical stunning. In parallel to a previous study investigating the stunning of carp [28], in the current pilot study, we analysed how stunning interventions were implemented on rainbow trout farms, which stunning method was applied and whether a stage of unconsciousness was achieved by the applied stunning intervention. As different electric current characteristics could be applied for electrical stunning, their stunning effect was assessed in a previous laboratory study.

## Results

### Laboratory study

#### Validation of indicators of consciousness

The loss of behavioural responses of fish to external stimuli or brainstem indicators of consciousness, like breathing movements of the gills, could be used for monitoring stunning success during routine commercial slaughter. In order to confirm whether the absence of behavioural traits/brainstem indicators was indeed correlated with a loss of consciousness, the occurrence of these indicators was validated against the recording of VER as a neurological measure for the perceptibility of external stimuli. Rainbow trout of marketable size were exposed to alternating or direct electric current of 50 V for 30 or 60 s. Before applying the electric current, all rainbow trout responded robustly to light flashes by VERs in the EEG (Table 1). When the electric current was applied, rainbow trout were motionless, the body muscles were contracted and breathing movements were discontinued. Immediately after the electric current had been switched off, all rainbow trout showed weakened muscle contraction and loss of the upright body posture. In some rainbow trout, trembling movements of the lower jaw (five out of 15 trout), irregular movements of the operculum (three out of 15 trout), irregular contractions of skeletal muscles and trembling of fins (one out of 15 trout) were observed (Table 1). In two fish, breathing movements and the vestibulo-ocular reflex (VOR) were present and these individuals recovered from stunning during subsequent observation (Table 1). From these individuals, VER were recorded immediately after the electric current flow had been switched off, while VER were not recorded after applying the electric current from the remaining rainbow trout (Table 1). VER were also not recorded from rainbow trout which exhibited trembling movements of the jaw or the fins or irregular contractions of skeletal muscles. These rainbow trout did not recover from stunning during the observation period of 10 min. Table 1 documents VER recordings and behavioural observations on the rainbow trout after electrical stunning for 30 and 60 s. On the basis of these observations, it was assumed that in rainbow trout, breathing movements and the VOR were lost at approximately the same time as VER. Thus, we assumed that rainbow trout are insensible when eye-rolling and breathing reflexes are absent. Therefore, during the following experiments and during the field study, these brainstem/behavioural indicators were used for assessing insensibility in trout after a stunning intervention.

**Table 1:** Correlation of electro-encephalogram recordings and behavioural/ brainstem indications in rainbow trout after electrical stunning

Electrical current	Number of rainbow trout						
	ac			dc			Summary
Duration of stun	total	30 s	60 s	Total	30 s	60 s	
VER before stunning	7	4	3	8	4	4	15/15
VER after stunning	2/7	2	0	1/8	1	0	3/15
Survival	2/7	2/4	0	1/8	1/4	0	3/15
OM	2/7	2/4	0	1/8	1/4	0	3/15
VOR	2/7	2/4	0	1/8	1/4	0	3/15
IOM	3/7	3/4	0	1/8	1/4	0	4/15
TFM	1/7	1/4	0	3/8	2/4	1/4	4/15
TJM	3/7	2/4	1/4	2/8	1/4	1/4	5/15
MC	0	0	0	1/8	1/4	0	1/15

*VER* visually-evoked responses, *OM* operculum movements, *VOR* vestibulo-ocular reflex, *IOM* irregular/ trembling movements of operculum, *TFM* trembling movements of fins, *TJM* trembling movements of lower jaw, *MC* irregular contractions of skeletal muscles

#### Stunning effect of different electric current characteristics

When an electric alternating current of 50 V was applied to rainbow trout, in four out of 189 specimens, VOR were present after the electric current had been switched off. These rainbow trout recovered from stunning. In the remaining specimens, however, VOR were not present and these rainbow trout did not recover from the stunning. The recovery rate was not different after stunning with AC frequencies of 50, 100 or 1000 Hz at different positions of the stunning electrodes nor when the electric current was applied for 30 or 60 s (Tables 2, and 3). When a DC voltage of 50 V was applied, in three out of 70 rainbow trout, VOR was observed after the electric current had been switched off and these three fish recovered from stunning (Tables 2, and 3). The remaining specimens did not show a VOR or OM and these individuals did not recover from stunning. The recovery rate was higher when DC electric current was applied for 30 s compared to a stunning duration for 60 s (Table 3).

**Table 2:** Electrical stunning of rainbow trout: electrical parameters and stunning success in the laboratory study

Electric current	Voltage [V]	Frequency [Hz]	Stun Duration [s]	Position of electrodes	Current density [A dm^−2^]	Stunning success [n VOR/n total number of fish]
AC	50	50	30	head/ tail	0.13 ± 0.002	2/14
	50	50	30	top/bottom	0.09 ± 0.001	0/10
	50	50	30	lateral	0.23 ± 0.003	0/10
	50	50	60	head/ tail	0.14 ± 0.003	0/14
	50	50	60	top/ bottom	0.09 ± 0.001	0/10
	50	50	60	lateral	0,24 ± 0.007	0/10
	50	100	30	head/ tail	0.14 ± 0.005	0/10
	50	100	30	top/bottom	0.10 ± 0.001	0/10
	50	100	30	lateral	0.23 ± 0.003	0/10
	50	100	60	head/ tail	0.14 ± 0.003	0/10
	50	100	60	top/ bottom	0.09 ± 0.001	0/10
	50	100	60	lateral	0,24 ± 0.007	0/10
	50	1000	30	head/ tail	0.14 ± 0.004	0/10
	50	1000	30	top/bottom	0.10 ± 0.001	1/10
	50	1000	30	lateral	0.24 ± 0.005	0/10
	50	1000	60	head/ tail	0.13 ± 0.003	0/11
	50	1000	60	top/ bottom	0.10 ± 0.002	1/10
	50	1000	60	lateral	0,25 ± 0.004	0/10
DC	50	n.a.	30	head/ tail	0.09 ± 0.008	1/16
	50	n.a.	30	top/ bottom	0.07 ± 0.003	1/10
	50	n.a.	30	lateral	0.18 ± 0.005	1/11
	50	n.a.	60	head/tail	0.11 ± 0.00	0/13
	50	n.a.	30	top/bottom	0.07 ± 0.00	0/10
	50	n.a.	60	lateral	0.18 ± 0.00	0/10

Conductivity of the water: 600 μS/cm; voltage: 50 V, given are mean ± standard deviation

*AC* alternating current, *DC* direct current; position of electrode: plate electrodes positioned at head and tail, top and bottom; lateral: at lateral sides of stunned rainbow trout

**Table 3:** Summary of electrical parameters and stunning process

Current	Frequency [Hz]	Stun duration [s]	Position of stunning electrodes	Stunning success [N VOR/ n total number of fish]
AC	50	–	–	2/68
	100	–	–	0/60
	1000	–	–	2/61
AC		30	–	3/94
		60	–	1/95
AC			Head/ tail	2/69
			Top/ bottom	2/60
			Lateral	0/60
Summary AC				4/189
DC		30		3/37*
		60		0/33
DC			Head/ tail	1/29
			Top/ bottom	1/20
			Lateral	1/21
Summary DC				3/70

*AC* alternating current, *DC* direct current, Statistical analysis: Kruskal Wallis ANOVA on ranks followed by Dunn’s post hoc test, AC, Frequency: *p* = 0.38, position of electrodes: p = 0.38, stun duration: *p* = 0.31, DC: plate position: *p* = 0.90, * statistically different to DC, 60 s at *p* = 0.026

In the experiments of the laboratory study on electrical stunning of rainbow trout, a current density of 0.06 to 0.25 A dm^− 2^ was achieved when an electric alternating or direct current at a voltage of 50 V was applied. In these experiments, 120 rainbow trout were treated with a current density below 0.1 A dm^− 2^ and 140 at a current density above 0.1A dm^− 2^. Of the 260 rainbow trout exposed to these electric currents, in seven specimens, a loss of consciousness was not achieved after applying electric current. Six of these rainbow trout were exposed to an electric current at a current density below 0.1 A dm^− 2^, between 0.06 and 0.09 A dm^− 2^, while the remaining rainbow trout was stunned at a current density of 0.18 A dm^− 2^. This indicates that a loss of consciousness could be more safely achieved after applying an electric current above 0.1 A dm^− 2^.

#### Physiological blood parameters in rainbow trout after electrical stunning

In order to monitor the stress level associated with the stunning of rainbow trout by electric currents of different characteristics, the haematocrit of circulating blood, as well as cortisol, potassium and sodium levels were measured in the blood or blood plasma of rainbow trout after stunning (Table 4 shows mean values). Haematocrit values of individual fish ranged from 16.0 to 61.0 L L^− 1^ (mean values per treatment varied from 39.0 to 49.0 L L^− 1^) and were significantly lower in rainbow trout stunned with AC frequencies of 100 Hz compared to the other types of electric currents used (Kruskal Wallis ANOVA on ranks, *p* < 0.05). Plasma cortisol levels in individual fish ranged from 2.1 to 201.8 ng mL^− 1^ (mean values per treatment: 17.9 to 68.0 ng mL^− 1^) and were significantly lower in rainbow trout stunned with DC for 30 s compared to those stunned with the other types of electric current (Kruskal Wallis ANOVA on ranks, *p* < 0.05). Potassium levels in individual fish ranged from 1.3 to 9.1 mmol L^− 1^ (mean values per treatment: 2.6 to 6.2 mmol L^− 1^) and were significantly lower in the blood of rainbow trout stunned with DC compared to those stunned with AC (Kruskal Wallis ANOVA on ranks, p < 0.05). Sodium levels in individual fish ranged between 111.0 and 185.0 mmol L^− 1^ (mean values per treatment: 150.0 to 168.0 mmol L^− 1^) and were similar in rainbow trout subjected to different types of electrical stunning currents (Kruskal Wallis ANOVA on ranks, *p* = 0.11, Table 4). Haemorrhages were detected in filets of 25–60% of the rainbow trout after electrical stunning. Haemorrhages were less frequent and less prominent in specimen stunned by DC compared to AC (Kruskal Wallis ANOVA on ranks, p < 0.05) currents. The presence of haemorrhages was not influenced by the position of the electrodes (*p* > 0.05), stunning duration (*p* = 0.06) or current frequency (Kruskal Wallis ANOVA on ranks, p = 0.1) when AC was used.

**Table 4:** Physiological parameters in blood of rainbow trout after stunning with different types of electric current

Current	Frequency [Hz]	Duration [sec]	Electrode position	Cortisol [ng ml^−1^]	Haematocrit [%]	Potassium [mmol l^−1^]	Sodium [mmol l^−1^]
DC		30	Head/ Tail	18.6 ± 14.78	43.2 ± 4.10	4.4 ± 0.95	167.0 ± 6.22
			Top/Bottom	21.7 ± 20.18	41.0 ± 8.82	5.7 ± 0.88	160.0 ± 5.06
			Lateral	29.5 ± 31.08	45.8 ± 9.75	4.6 ± 0.50	152.0 ± 18.43
		60	Head/ Tail	17.9 ± 14.26	42.8 ± 6.20	4.6 ± 0.58	163.0 ± 5.30
			Top/Bottom	74.6 ± 54.26	49.0 ± 7.21	5.2 ± 0.58	166.0 ± 6.94
			Lateral	41.4 ± 51.22	37.9 ± 12.39	5.2 ± 1.12	159.0 ± 8.00
AC	50	30	Head/ Tail	42.0 ± 15.56	43.4 ± 3.46	3.7 ± 1.11	163.0 ± 7.11
			Top/Bottom	54.0 ± 29.69	48.5 ± 5.24	5.4 ± 1.08	164.0 ± 6.39
			Lateral	38.0 ± 20.51	39.9 ± 4.42	4.4 ± 0.85	162.0 ± 2.75
		60	Head/ Tail	57.0 ± 25.53	47.8 ± 4.62	4.6 ± 1.04	157.0 ± 8.72
			Top/Bottom	82.0 ± 62.44	42.9 ± 5.11	4.3 ± 0.53	156.0 ± 3.68
			Lateral	73.0 ± 60.90	47.1 ± 4.32	4.2 ± 0.52	150.0 ± 15.83
	100	30	Head/ Tail	74.0 ± 31.13	44.6 ± 3.36	6.2 ± 2.21	167.0 ± 7.69
			Top/Bottom	41.0 ± 38.77	44.3 ± 2.71	4.3 ± 0.63	168.0 ± 5.95
			Lateral	59.0 ± 24.11	39.5 ± 3.89	4.8 ± 1.51	158.0 ± 10.03
		60	Head/ Tail	68.0 ± 36.0	39.5 ± 4.57	4.4 ± 0.68	165.0 ± 1.77
			Top/Bottom	50.0 ± 25.55	42.9 ± 3.72	4.3 ± 0.94	164.0 ± 5.31
			Lateral	24.0 ± 18.53	39.0 ± 6.74	5.1 ± 1.26	158.0 ± 3.54
	1000	30	Head/ Tail	51.0 ± 3878	48.3 ± 10.00	3.5 ± 2.01	160.0 ± 16.22
			Top/Bottom	60.0 ± 44.11	40.1 ± 3.83	4.0 ± 1.40	1620 ± 5.51
			Lateral	40.0 ± 23.25	45.3 ± 4.43	3.0 ± 0.48	163.0 ± 5.42
		60	Head/ Tail	37.0 ± 24.96	41.9 ± 5.08	3.4 ± 0.60	167.0 ± 3.07
			Top/Bottom	65.0 ± 57.09	43.0 ± 4.34	2.6 ± 0.61	167.0 ± 4.93
			Lateral	37.0 ± 25.53	39.9 ± 4.82	5.3 ± 1.62	164.0 ± 3.72

Shown are mean ± standard deviation of n = 10–12 specimens per treatment. Electrode position: plate electrodes positioned at head and tail, top and bottom; or lateral: at lateral sides of stunned rainbow trout

Statistical analysis: Kruskal Wallis ANOVA on ranks followed by Dunn’s post hoc test. Cortisol, haematocrit, sodium: no differences at p < 0.05. Potassium: AC 1000 HZ, lateral 30 s and1000 Hz top/bottom 60 were significantly different to the other groups at p < 0.05

### Field study

In most of the analysed conventional production chains, rainbow trout were stunned by manually applied percussion (eight stunning interventions, Table 5) or by electrical stunning in a water bath (wet electrical stunning; nine stunning interventions, Table 5). One farm used electrical stunning after removing the rainbow trout from the water and placing them in a metal tank. On one farm, the rainbow trout were placed outside the water on a device with two belts serving as negative and positive electrodes (dry electrical stunning) before putting the fish in a stunning tank filled with water. An overview of the stunning procedures is provided in Table 5. On three farms, manual percussion was applied after electrically stunning rainbow trout. For electrical stunning, the farmers used commercially available or self-modified stunning devices (Table 5). After percussive stunning, 92.2% (130 out of 141 individuals, Table 5) displayed no behavioural indicators of consciousness and after electrical stunning, 83.3% (125 out of 150 specimens, Table 5) showed no behavioural/brainstem indicators of consciousness. The stunning success varied between farms. On most farms on which rainbow trout were stunned by percussion or electrical current, all fish were successfully stunned. Only on one farm were a mere 16 specimens out of a total of 20 successfully stunned by percussion (Table 5). On three farms, which used electrical stunning, just two out of ten or 14 out of 20 rainbow trout did not display any signs of consciousness after the electric current had been applied. Rainbow trout removed from the water and subsequently stunned by dry electrical stunning or electrical stunning followed by percussion, displayed no behavioural indicators of consciousness (Table 5).

**Table 5:** Stunning and killing of rainbow trout in the field study: overview of applied stunning methods, electrical parameters, stunning success and times

Stunning intervention.	Electrical parameters				Stunning success	Injuries/ mis-hits	Time until exsanguination	Behavioural signs at time of slaughtering
	Water conductivity [μS/cm; MIN-MAX]	Voltage [V; MIN-MAX]	Electric current densities [A/dm^**2**^; MIN-MAX]	Stunning time (seconds or minutes)	[%; (n/total number of fish)]	[%; (n/total number of fish)]	[seconds or minutes; MIN-MAX]	[%; (n/total number of fish)]
**Stunning by percussion (*n*** **= 8 stunning interventions)**								
1	n.a.	n.a.	n.a.	n.a.	83.0 (7/11)	0.0 (0/11)	60–95 s.	27.0 (3/11)
2	n.a	n.a	n.a	n.a	95.0 (19/20)	50.0 (4/8)	15–20 s.	0.0 (0/20)
3	n.a	n.a	n.a	n.a	95.0 (19/20)	70.0 (7/10)	15–30 min.	5.0 (1/20)
4	n.a.	n.a	n.a	n.a.	100.0 (20/20)	10.0 (1/10)	5–10 min.	0.0 (0/20)
5	n.a.	n.a.	n.a.	n.a.	100.0 (20/20)	10.0 (1/10)	20 s. – 5 min.	0.0 (0/20)
6	n.a.	n.a.	n.a.	n.a.	80.0 (16/20)	50.0 (5/10)	10–20 min.	10.0 (2/20)
7	n.a.	n.a.	n.a.	n.a.	100.0 (10/10)	100.0 (10/10)	2–5 min.	0.0 (0/10)
8	n.a.	n.a.	n.a.	n.a.	95.0 (19/20)	50.0 (5/10)	20–25 min.	10.0 (1/10)
**Summary Percussion (N = 8)**	**n.a**	**n.a**	**n.a**	**n.a**	**92.2 (130/141)**	**41.8 (33/79)**	**15 s. – 30 min.**	**5.3 (7/131)**
**Electrical stunning (**^**a**^**: in a water bath,*n*** **= 9 stunning interventions;**^**b**^**: on wet electrodes outside the water, n = 1 stunning intervention)**								
9^a^	453	36–47	0.0163–0.0213	3 min.	100.0 (10/10)	10.0 (1/10)	2–5 min.	0.0 (0/10)
10^a^	334	33–36	0.0245–0.0267	1 min.	70.0 (14/20)	60.0 (6/10)	60–90 s.	35.0 (7/20)
11^a^	613	36	0.0061	30–45 s.	100.0 (10/10)	0.0 (0/10)	10–120 min.	0.0 (0/10)
12^a^	604	36	0.0064	30–45 s.	100.0 (10/10)	0.0 (0/10)	10–120 min.	0.0 (0/10)
13^a^	438	34	0.0196	60 s.	100.0 (20/20)	20.0 (2/10)	20–90 min	10.0 (1/10)
14^a^	969	20	0.121	20 s.	20.0 (2/10)	30.0 (3/10)	2–4 min.	80.0 (8/10)
15^a^	910	20	0.114	20 s.	20.0 (2/10)	30.0 (3/10)	2–4 min.	70.0 (7/10)
16^a^	756	230	0.435	30 s.	95.0 (19/20)	20.0 (2/10)	2 min.	10.0 (1/10)
17^a^	756	230	0.435	30 s.	95.0 (19/20)	40.0 (4/10)	2 min.	10.0 (1/10)
19^b^	209	53.3	Not measurable	2 min.	95.0 (19/20)	70.0 (7/10)	2–5 min.	10.0 (1/10)
**Summary Electrical stunning (N = 10)**	**334–969**	**20–230**	**0.0061–0.435**	**20 s. – 3 min.**	**83.3 (125/150)**	**28.0 (28/100)**	**1–120 min.**	**23.6 (26/110)**
**Electrical stunning followed by percussion (**^**c**^**: electrical stunning in a water bath, n = 2;**^**d**^**: electrical stunning first on wet electrodes outside the water followed by electrical stunning in a water bath, n = 1)**								
20^c^	577	37.6	0.0434	20 s.	100.0 (10/10)	20.0 (2/10)	20 s.	0.0 (0/10)
21^c^	822	28.5	0.146	1 min.	100.0 (6/6)	0.0 (0/6)	60–90 s.	0.0 (0/6)
22^d^	441	36	0.1359	8 min (3 min dry, 5 min in water)	100.0 (20/20)	0.0 (0/10)	2–5 min.	0.0 (0/10)
**Summary Electrical stunning followed by percussion** **(N = 3)**	**577–822**	**28.5–37.6**	**0.0434–0.146**	**20 s. – 8 min.**	**100.0 (26/26)**	**12.5 (2/16)**	**20 s. – 5 min.**	**0 (0/16)**

During electrical stunning, the stunning devices generated an electrical voltage of between 20 and 230 V, which resulted in electric current densities of between 0.0061 and 0.435 A dm^− 2^. The electric current was applied for a duration of 20 s up to approx. 3 min. When the current density or conductivity of the water were low or the electric current was applied for just a few seconds, the respective percentage of successfully stunned rainbow trout was low (Table 5). Low current density was mainly measured on farms where the electric current was applied to water with a low conductivity of approx. 330 μS cm^− 1^. Hence, the stunning success was significantly influenced by the conductivity of the water (*p* = 0.01) and the duration of the stun (*p* = 0.04, multiple linear regression). When on these farms the water conductivity in the stunning tank was elevated by adding salt to above 500 μS cm^− 1^, all rainbow trout were successfully stunned. In addition, when on farms which applied the electric current for a few seconds only, the stunning time was extended to more than 30 s, all rainbow trout were stunned successfully (data not shown). On most of the farms, the rainbow trout were killed within 2–5 min after stunning, mainly by evisceration or by gill cuts. On some farms however, the time period from stunning until killing took up to 120 min (Table 5).

**Fig. 1:**
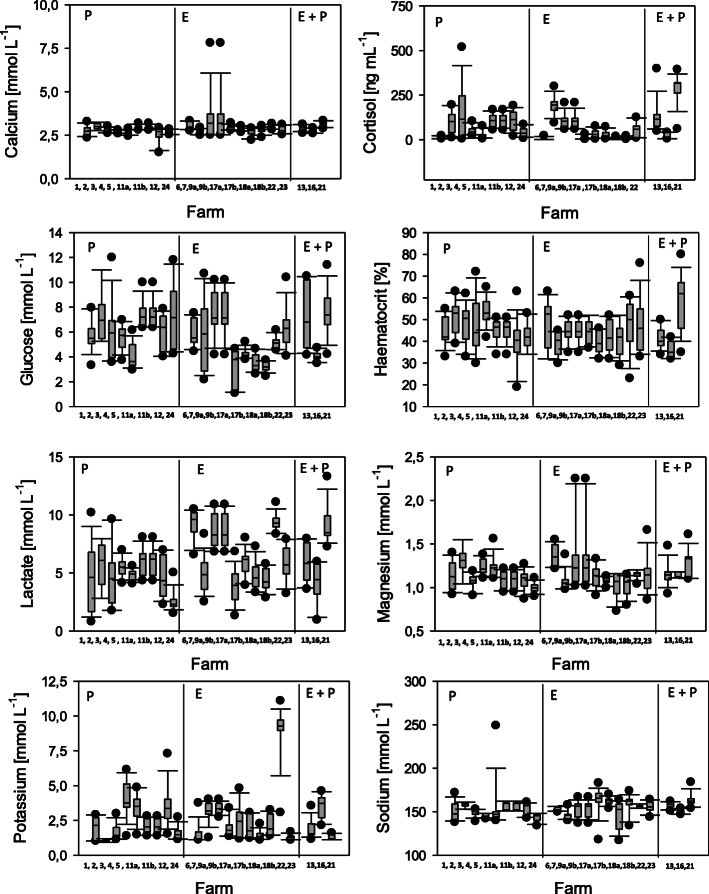
Physiological parameters in the blood of rainbow trout after different stunning methods during commercial production lines. E: Electrical stunning, E + P: Electrical stunning followed by percussion, P: stunning by percussion. 1–23: number of trout farms where the stunning process was analysed. Shown are median (horizontal line), 25–75 percentiles (boxes), 5–95 percentiles (whiskers) and outliers of measurements from *n* = 10 specimens per farm

**Fig. 2:**
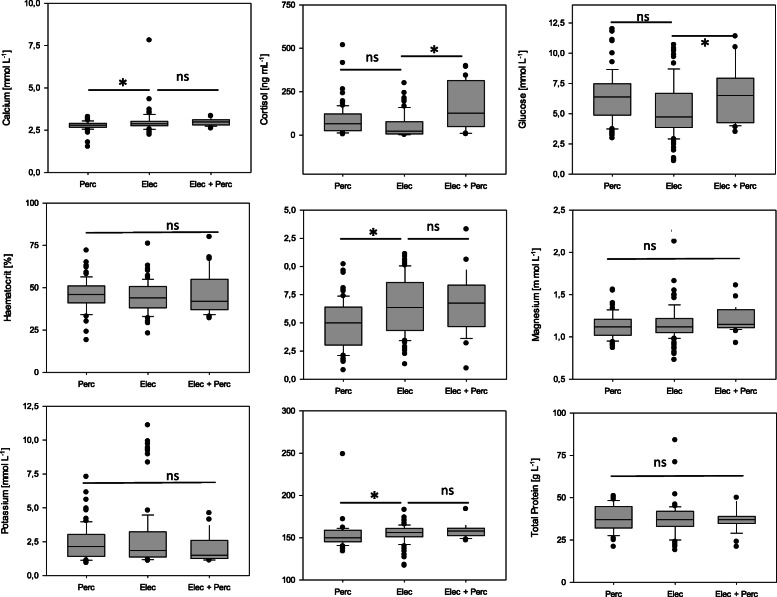
Effect of different stunning interventions on physiological blood parameters of rainbow trout during commercial slaughter on German trout farms. Elec: Electrical stunning, Elec + Perc: Electrical stunning followed by percussion, Perc: Stunning by percussion. Shown are median (horizontal line), 25–75 percentiles (boxes), 5–95 percentiles (whiskers) and outliers of measurements from *n* = 30 (Elec + Perc), 85 (Perc), and 100 (Elec) specimens per stunning intervention. *: statistical significant differences at *p* < 0.05%, ns: differences at *p* > 0.05% after mixed model analysis of variance

In order to monitor stress levels of rainbow trout in the different production chains on the visited aquaculture farms, haematological parameters including haematocrit, cortisol, glucose, lactate and potassium and sodium levels were measured in blood or blood plasma and compared for the applied stunning methods (Figs. 1, 2). Mean plasma concentrations of the bivalent ions calcium and magnesium ranged between 2.6 and 4.3 mmol L^− 1^ (Ca^2+^) and 1.0 and 1.4 mmol L^− 1^ (Mg^2+^) with only minor differences between production chains and stunning protocols. Significant variation was observed in cortisol levels between production chains, and mean plasma cortisol measurements varied between 5.1 and 311.1 ng L^− 1^. Due to the large variation in the cortisol levels between farms, significant differences were not observed between production chains with percussive stunning or electrical stunning (mixed model analysis of variance, *p* = 0.13). However, rainbow trout which were subjected to electrical stunning followed by percussion had significantly elevated plasma cortisol levels compared to the other stunning protocols (mixed model analysis of variance, *p* < 0.001). Glucose levels varied to a lesser extent and ranged between 3.1 and 7.5 mmol L^− 1^ with significantly lower values after electrical stunning compared to percussive stunning (mixed model analysis of variance, *p* = 0.004) or electrical stunning followed by percussion (mixed model analysis of variance, *p* = 0.022). Haematocrit values ranged from 36.2 to 53.6% and were not significantly different in rainbow trout subjected to percussive stunning or stunning by electrical current (mixed model analysis of variance, *p* = 0.40). Mean lactate levels per farm ranged from 3.8 to 9.3 mmol L^− 1^ and were significantly elevated in the blood of rainbow trout subjected to electrical stunning (mixed model analysis of variance, *p* < 0.0001) or electrical stunning followed by percussion (mixed model analysis of variance, *p* = 0.003) compared to stunning by percussion. Mean plasma sodium levels ranged from 143.2 to 163.6 mmol L^− 1^ and were significantly lower in rainbow trout stunned by percussion (mixed model analysis of variance, *p* = 0.027) compared to other stunning procedures. A direct correlation between insufficient stunning and elevated cortisol (multiple linear regression, *p* = 0.748) and/or glucose (multiple linear regression, *p* = 0.09) levels or decreased sodium (multiple linear regression, *p* = 0.21) measurements could not be registered.

## Discussion

The cognitive and mental capacities of fish as a basis for their perception of fear and pain are now widely accepted among scientists [6, 10, 11]. Cognitive capacities of fish were studied in several species (see [6, 10, 11]). These studies showed that several teleost fish species respond to stressful situations by behavioural and physiological reactions [9, 13]. In addition, fish, being vertebrates, are also included in EU and national legislation on the protection of welfare of farmed animals [19–21]. These regulations stipulate that prior to killing or slaughter, fin fish have to be stunned using higher welfare methods like percussion and electrical stunning. A considerable amount of rainbow trout produced in the EU in freshwater are raised by family businesses with an annual production of less than 20 metric tonnes of portion-size trout and are marketed directly [4, 22]. These fish are killed on site and not in a processing plant. Prior to the present study, no substantial data are available on the stunning and killing methods used by these small businesses and about the stunning success of the applied methods. The present survey gives a first insight into how stunning interventions are implemented in direct marketing of rainbow trout even though a limited number of farms were visited. In Germany, the application of percussive or electrical stunning is stipulated by the German Regulations for Animal Welfare during Slaughter [21] and therefore one of these methods or a combination of both was applied on all the trout farms visited during the present survey. On most of the farms, these stunning interventions resulted in effective stunning and no behavioural signs of consciousness were observed at the time of slaughtering, irrespective of whether the trout had been stunned by percussion, electrical stunning or by a combination of both methods. However, on some farms, behavioural indicators of consciousness could be observed in several of the fish after the stunning intervention and even at the time of slaughtering. On several farms, the slaughter protocols needed to be adjusted in respect of current density and stun duration. In addition, this clearly documents the importance of adequately training operators in the proper application of the stunning method used on farms and in recognising whether fish are insensible or not (see Ashley et al. [29]).

On farms, stunning success can routinely only be assessed by observing behavioural indicators of consciousness like the brain stem indicators opercular movements (indicating breathing activity) or the eye-rolling reflex. Data from the present laboratory study document that in rainbow trout, electrical responses of the brain to light stimuli were lost at the same time as the breathing and eye-rolling reflexes. This confirms previous observations on rainbow trout, which showed epileptiform brain activity in EEG recordings after electrical stunning interventions. At the same time, the brainstem indicators breathing and eye-rolling reflex were not present [26, 27]. On the basis of these observations, it can be concluded that the absence of behavioural/brainstem indicators correlates with neuronal indicators of insensibility like abnormal EEG or the absence of VER of the brain to light stimuli and therefore these indicators can be used for monitoring the stunning success on farms. In the present laboratory study, insensible rainbow trout were not completely motionless but often showed irregular contractions of skeletal muscles, trembling movements of the lower jaw, fins, or the operculum during several minutes after the stunning intervention. Therefore, the presence of irregular contractions of skeletal muscles or trembling movements of the jaw cannot be used to recognise consciousness in rainbow trout.

In Germany, electrical stunning is stipulated for fish by “German regulation for animal welfare during slaughter” but electrical parameters are not defined except for the stunning of eels [21]. Our results suggest electrical stunning of portion sized rainbow trout could be achieved by using direct (DC) as well as alternating currents (AC) at frequencies of 50, 100 or 1000 Hz. In a previous study [30], rainbow trout were stunned with alternating currents between 50 and 2000 Hz for 5 s, and the recovery time of fish decreased with increasing current frequency. The apparent stunning time for 2000 Hz was very short [30]. In the present study, electric currents were applied for 30 or 60 s as recommended by [31], and in the vast majority of fish a permanent insensibility was achieved. In our laboratory and field studies, effective stunning was more safely achieved when rainbow trout were stunned at a current density above 0.1 A dm^− 2^ (see also [25]). This was particularly evident during the field study, where a significant proportion of fish were not successfully stunned on farms which applied a low current density or a current for only a short period of time. The current density is influenced by water conductivity [31], which in freshwater may vary on a broad scale. On the farms visited during the current field study, water conductivity varied between 334 and 969 μS cm^− 1^, which significantly influenced electric field strength and stunning success. The field study gave evidence that the stunning success was improved at water conductivity above 500 and below 1000 μS cm^-1.^ Thus, it can be recommended to adjust water conductivity in the stunning tank accordingly, for instance by adding salt.

In the blood of rainbow trout from the laboratory study as well as from the field study, plasma electrolytes, cortisol, glucose and lactate levels were measured as possible indicators for stress during the stunning and slaughter process. During the laboratory study, the measurements for haematocrit, potassium, sodium, and in particular plasma cortisol levels varied to a great extent between individuals. When particular stunning parameters were considered, lower cortisol levels were observed in trout stunned by direct current for 30 s compared to those stunned with other types of electric currents. In addition, lower potassium levels were measured in trout stunned by direct current compared to those stunned by alternate current. In the field study, the measurements of haematological parameters of rainbow trout varied to a large extent between farms. This was particularly observed in plasma cortisol and to a lesser extent in glucose, lactate and sodium. When the different stunning procedures were compared, in particular rainbow trout, stunned by dry electrical stunning, had elevated plasma cortisol, glucose and lactate levels and decreased plasma sodium levels, which might indicate an increased stress response in these fish, most likely due to their exposure to air during the stunning process. This was already addressed in the EFSA report on the stunning of trout [22]. A direct correlation between stunning success on individual farms and elevated plasma cortisol or glucose levels could not be documented. During the pre-slaughter process, also the handling of rainbow trout differed largely between farms. On some farms, the process of harvesting the fish from the pond, transportation on site, and stunning and killing was streamlined and was performed with minimal disturbance to the trout, while on other farms trout were held under crowded conditions for prolonged periods of time prior to stunning or transportation. The wide variation in the measurements of cortisol, glucose or sodium between farms suggests that these parameters were largely influenced by hazards related to pre-slaughter conditions of trout in addition to the stunning process.

## Conclusion

The current laboratory study shows that trout can safely be stunned by electrical stunning using both direct and alternate electric current of different frequencies. For effective stunning of rainbow trout, associated with a stage of insensibility, an electric current at a density of at least 0.1 A dm^− 2^ should be applied. As the electric current density is largely influenced by water conductivity, this field strength is reached best at a water conductivity above 500 and below 1000 μS cm^− 1^.The stage of consciousness can be monitored by the observation of brainstem/behavioural indicators, in particular, breathing and eye-rolling reflexes. When these reflexes were absent, VER was also not be recorded from the brain of the trout in response to light stimuli, which indicated that the brain of these fish was not responding to external stimuli. The current field study indicated that effective stunning interventions can be implemented in production chains of conventional rainbow trout aquaculture. On farms, an immediate stunning of all processed rainbow trout was achieved with percussive as well as with electrical stunning. However, the field study revealed that the stunning process on farms requires careful attention. The operators need to be trained in how to adequately use the stunning devices and they have to be aware of indicators of consciousness such as regular opercular movements or the eye-rolling reflex. Rainbow trout need to be re-stunned by percussion as soon as these indicators are visible. Because fish must remain unconscious until death, the time between stunning and killing by gill cut or evisceration as part of processing need to be as short as possible. In addition, farmers have to be aware of hazards during the pre-slaughter process of the fish which also might impair the welfare of rainbow trout at the time of stunning and killing.

## Methods

### Laboratory study

A laboratory study was conducted to analyse whether after electrical stunning of rainbow trout, a loss of behavioural / brainstem indicators of consciousness, such as operculum movements or eye-rolling reflex correlates with the loss of VER as evidence of unconsciousness. Subsequently, it was evaluated whether a loss of consciousness was also achieved after applying an electric field at different alternating current (AC) frequencies and different field orientations.

#### Fish

Rainbow trout, *Oncorhynchus mykiss* (*n* = 274) with a size ranging from 25.0–33.5 cm and weighing 211.3–570.9 g were collected from a rainbow trout farm in the vicinity of Hannover, Germany and kept in groups of five-ten individuals in 250 L flow-through tanks in aerated dechlorinated tap water at 10–15 °C for up to one week. All rainbow trout were not showing any clinical signs of disease. All experiments were performed in accordance with internationally accepted veterinary standards and federal guidelines for the protection of animals during experimentation after approval by the Lower Saxony State Office for Consumer Protection and Food Safety; Germany (LAVES Oldenburg, reference number 06/1144). The experiments were performed in a consecutive manner. Only one stunning intervention was applied to an individual rainbow trout. Rainbow trout were allocated to the different experimental groups on the basis of a randomized list.

#### Recording of visual-evoked responses (VER) of the brain

For recording VER, EEG electrodes were positioned intracranially over the optic tectum and the *cerebellum* of rainbow trout as described elsewhere [28]. Briefly, the recording electrode was positioned over the right hemisphere of the optic tectum, and two other wire electrodes over the left hemisphere of the *optic tectum* and the *cerebellum,* respectively*,* were used to suppress current artefacts and for grounding. Prior to implanting the electrodes, rainbow trout were anaesthetised in a solution of buffered MS222 (150 mg L^− 1^; Pharmaq Ltd., UK) and the narcosis was maintained during the operation by constantly applying water with buffered MS 222 (100 mg L^− 1^) to the gills of the rainbow trout in a closed water circuit system. The electrodes were fixed to the skull of the rainbow trout by means of the bonding agent GLUMA comfort bond (Kulzer GmbH, Germany) as described by [28].

**Fig. 3:**
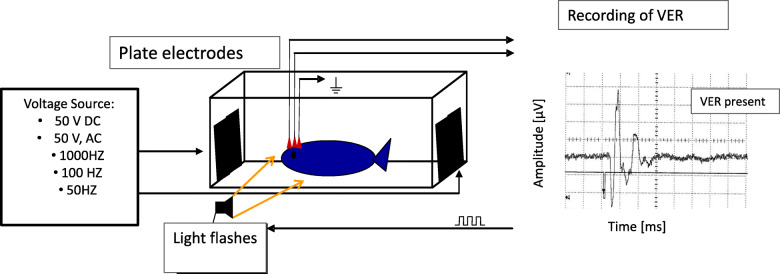
Experimental set up for recording visually-evoked responses of the brain to light flushes after electrical stunning interventions

In order to generate visually-evoked responses of the brain, white light flashes were presented by means of a stroboscope (eurolite Action Strobe, Conrad Electronic GmbH & Co KG, Germany), which was triggered by a pulse generator (HSE Stimulator P, Harvard Apparatus Ltd., UK). Electro-encephalic responses to the light stimuli were recorded and amplified as described by [28] using a differential amplifier (DAM 50, World Precision Instruments, LLC, USA, band pass filter 10 Hz to 3 kHz, 1000-fold amplification), a downstream bandpass filter (Ithaco Electronic Filter 4213, DL Instruments LLC, USA) and a digital oscilloscope/ averager (VC 7504, Hitachi Europe GmbH, Germany). By means of the oscilloscope, 512 recorded signal cycles of 116 ms duration were averaged and visualised (Fig. 3). The trigger signal was displayed on the second channel of the oscilloscope for a temporal correlation of the VER with the light stimuli. The experimental room was kept dark during presentation of light stimuli and recording of VERs.

#### Validation of behavioural indicators of consciousness against VER measurements

**Fig. 4:**
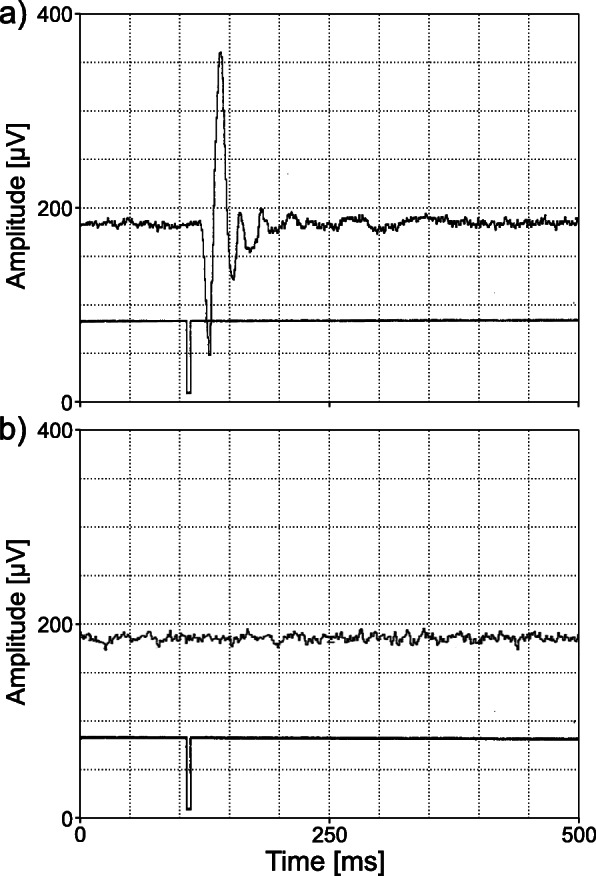
Averaged visually-evoked response (VER, *n* = 512 signals) in the electro-encephalogram of rainbow trout. Upper line: electro-encephalogram of a rainbow trout, lower line: trigger pulse. **a** neuronal responses were triggered by light flashes. The onset of the VER corresponds to the trigger pulse. **b** averaged control condition without light stimulus. Grid: 50 ms and 50 μV, respectively

All rainbow trout used for validating behavioural/ brainstem indicators of consciousness against VER recordings were individually equipped with EEG electrodes and placed in a fenestrated PVC aquarium with plastic grids and with internal adjustable separators in order to restrain possible movements of the rainbow trout which could result in a disconnection of recording electrodes. Then, the restrained rainbow trout were placed individually in a plastic tank equipped with plate electrodes for applying electrical stunning. The stunning tank was filled with 130 L water at a temperature of 12–15 °C and a water conductivity of 600 μS cm^− 1^. The stunning electrodes were stainless-steel plate electrodes, each having an area of 2.72 dm^2,^ being placed at a distance of 54.0–54.5 cm at opposite sides of the tank towards the head and tail of the rainbow trout. Prior to applying electrical stunning, rainbow trout were observed for an upright body posture and regular opercular movements as indicators for consciousness. Then, EEG recordings were taken with and without light flashes to confirm the correct position of the electrodes (Fig. 4) and whether recorded signals were indeed visually-evoked responses of the rainbow trout’s brain. Thereafter, the experimental rainbow trout was disconnected from the EEG devices and the plate electrodes were connected to a power supply delivering a sinusoidal AC electric current at 50 V and an adjustable frequency of 50 to 1000 Hz or to a device generating an electric direct current (DC) voltage of 50 V (both instruments were constructed by the technical service team at LAVES in order to mimic various stunning devices present on fish farms). The applied voltages and currents were measured with a multimeter (Voltcraft DC 170 Digital Multimeter, Conrad Electronic GmbH & Co KG, Germany) and achieved current densities were calculated by using the formula: current density [A dm^− 2^] = conductivity of the water [S dm^− 2^] * voltage [v] / distance of plate electrodes [dm].

Electric alternating current at 50 Hz or DC voltage, both at 50 V, was applied for 30 or 60 s. After stunning, the connection to the EEG recording instruments was immediately re-installed and EEG responses of the fish to light stimuli were recorded. First, VER were registered 30 s post stunning and the recordings were continued until 10 min post stunning. During this period of time, the rainbow trout were observed for the presence or absence of behavioural traits including irregular/ trembling movements of the operculum (IOM), periodic opercular movements (OM) as an indicator of breathing activity, irregular contractions of skeletal muscles (MC), trembling movements of fins (TFM), trembling movements of the lower jaw (TJM), and eye rolling/ vestibulo- ocular reflex (VOR). In general, rainbow trout died during a 10-min period of unconsciousness.

#### Assessment of the stunning effect of an electric field at different frequencies and of different orientations

In a further experiment, it was investigated whether a loss of consciousness was induced in rainbow trout after stunning with an electric alternate current at different frequencies and of different field orientations. For this, rainbow trout were placed in the fenestrated plastic aquarium, restrained in order to avoid unwanted movements and placed in the stunning tank as described above. To apply the electric current, stainless-steel plate electrodes were placed at opposite sides of the tank. Electrodes positioned towards the head and tails of the rainbow trout had an area of 2.72 dm^2^ each and were placed at a distance of 54.0–54.5 cm, electrodes with an area of 5.06 dm^2^ were positioned laterally of the rainbow trout at a distance of 26.1–26.6 cm and electrodes having an area of 22.33 dm^2^ were placed at a distance of 66.3–67.0 cm at the top and bottom of the stunning tank. An electric DC voltage of 50 V or an AC voltage of 50 V at a frequency of 50, 100 or 1000 Hz was applied for 30 and 60 s. The stunning success was assessed by monitoring the brainstem/ behavioural indicators periodic opercular movements, vestibulo-ocular/ eye-rolling reflex and righting behaviour over a period of 10 min post stunning.

Thereafter, blood samples were collected from the caudal vein of the rainbow trout and the haematocrit was determined as previously described [27]. From a portion of the blood sample, the blood plasma was separated and cortisol, potassium and sodium levels were determined as described by [27]. Subsequently, the carcass was filleted and examined for the presence of haemorrhages next to the vertebral column and in the fillet.

### Field study

To monitor the effectivity of stunning procedures applied in different production chains of rainbow trout in Germany, on 18 rainbow trout farms located in the German federal states of Bavaria, Lower Saxony, North Rhine-Westphalia and Saxony, the processes of stunning and slaughtering were analysed during routine marketing operations. Some farms used different methods for stunning and killing and therefore, in total, 21 processes for stunning and killing were analysed. For each process, the applied stunning method was registered. In the case of electrical stunning, electric conductivity of the water, distance between the electrodes, electric current density and stunning duration were recorded as further relevant parameters. Directly after stunning, behavioural responses were monitored and the percentage of fish showing brainstem/behavioural indicators of consciousness and reflexes was registered. When electrical stunning was applied, the percentage of rainbow trout with externally visible injuries was documented and in the case of percussive stunning, the percentage of trout experiencing mishits (when the stroke hit the skull but not the brain region) was recorded. When percussive stunning was used as a stunning method, one or two quick blows to the skull were applied manually to each carp with a self-manufactured wooden or plastic club. For electrical stunning, small groups of trout were placed into a plastic tank with plate electrodes (manufactured by FIAP, Germany; AKG, Germany; Karl von Keitz, Germany, or were self –manufactured). For dry electrical stunning, the rainbow trout were placed outside the water on a self-manufactured device with two belts serving as negative and positive electrodes before putting the fish in a stunning tank filled with water. Finally, the time-span between stunning and slaughtering the rainbow trout by exsanguination and/or evisceration was noted.

Furthermore, on each farm, immediately after stunning, blood samples were collected from the caudal vein of six to 11 rainbow trout into syringes with lithium-heparinised beads (Sarstedt GmbH, Germany) to prevent blood clotting. From the blood, the haematocrit was determined immediately by centrifuging a blood sample in a haematocrit centrifuge. In addition, a part of the blood sample was centrifuged at 600 x *g* at 4 °C for 15 min; the supernatant plasma was collected and transported on ice to the laboratory. There it was frozen at − 80 °C and kept for further use. From blood plasma samples, the blood cortisol level was determined by solid phase enzyme-linked immunosorbent assay (ELISA RE52611, IBL International GmbH, Germany). Calcium, glucose, lactate, magnesium, potassium, sodium and total protein concentrations were determined with an automated blood analyser (ABX Penta 400, Horiba Ltd., Kyoto, Japan, operated by the clinical chemical laboratory at the Clinic for Cattle at the University of Veterinary Medicine, Hannover). The carcass was filleted and examined of the presence of haemorrhages or blood spots in the fillet.

### Statistical analysis

The data were tested for normality and homoscedasticity with a Shaprio-Wilk Test and by computing a Spearman’s rank correlation between the absolute values of the residuals and the observed value of the independent value at *p* < 0.05. When variances were considered equal and the data were distributed normally, an ANOVA, followed by Tuckey’s post-hoc test were used to detect statistically significant differences between different stunning protocols. When the test for normality failed, the data were analysed using the Kruskal-Wallis ANOVA on ranks test followed by Dunn’s post-hoc test.

In addition, measurements of the blood parameters were analysed for differences between stunning procedures with a mixed model analysis of variance. In this analysis, fish specimen within a farm were considered as g-side random effects in the model (variance-components), taking into account that fish within a farm are subject to more similar conditions than between farms. Proc Glimmix was used to calculate the linear Model. The statistical evaluation of the mixed linear model was carried out using SAS 9.4 m5 with the Enterprise Guide Client 7.15 (SAS Institute Inc., Cary, NC, USA). A multiple linear regression was calculated in order to analyse the effects of current density, water conductivity and duration of stunning on stunning success and the effect of the different stunning procedures on cortisol, glucose or sodium measurements. In all analyses, differences between groups were considered significant at p < 0.05.

## Data Availability

The data collected and analysed during the current study are available from the corresponding author on reasonable request.

## Abbreviations

A: Ampere
AC: Alternating electrical current
ANOVA: Analysis of variance
°C: Degree Celsius
Ca^2+^: Calcium ion
cm: Centimeter
CO_2_: Carbon dioxide
DC: Direct electrical current
dm: Decimeter
EC: European council
EEG: Electro encephalogram
EFSA: European Food Safety Agency
ELISA: Enzyme linked immunosorbent assay
EU: European Union
g: Gram
Hz: Hertz
IOM: Irregular movement of the operculum
L: Liter
LAVES: Lower Saxony State Office for Consumer Protection and Food Safety
MC: Contractions of the skeletal muscles
Mg^2+^: Magnesium ion
min: Minute
mL: Milliliter
mmol: Milli mol
μS: Micro Siemens
ng: Nanogram
OM: Opercular movements
p: Probability of error
s: Second
SME: Small and medium enterprise
TFM: Trembling movements of fin
TJM: Trembling movement of jaw
UK: United Kingdom
V: Volt
VER: Visually evoked response
VOR: Vestibule-ocular reflex
